# Dorsal skinfold chamber models in mice

**DOI:** 10.3205/iprs000112

**Published:** 2017-07-10

**Authors:** Jeannine Schreiter, Sophia Meyer, Christian Schmidt, Ronny M. Schulz, Stefan Langer

**Affiliations:** 1 Department of Plastic, Aesthetic and Special Hand Surgery, Clinic and Polyclinic for Orthopaedics, Traumatology and Plastic Surgery, University Hospital Leipzig, Germany; 2 Centre for Biotechnology and Biomedicine, Leipzig, Germany

**Keywords:** angiogenesis, dorsal skinfold chamber, fat graft, intravital fluorescence microscopy

## Abstract

**Background/purpose**: The use of dorsal skinfold chamber models has substantially improved the understanding of micro-vascularisation in pathophysiology over the last eight decades. It allows *in vivo* pathophysiological studies of vascularisation over a continuous period of time. The dorsal skinfold chamber is an attractive technique for monitoring the vascularisation of autologous or allogenic transplants, wound healing, tumorigenesis and compatibility of biomaterial implants. To further reduce the animals’ discomfort while carrying the dorsal skinfold chamber, we developed a smaller chamber (the Leipzig Dorsal Skinfold Chamber) and summarized the commercial available chamber models. In addition we compared our model to the common chamber.

**Methods:** The Leipzig Dorsal Skinfold Chamber was applied to 66 C57Bl/6 female mice with a mean weight of 22 g. Angiogenesis within the dorsal skinfold chamber was evaluated after injection of fluorescein isothiocyanate dextran with an Axio Scope microscope. The mean vessel density within the dorsal skinfold chamber was assessed over a period of 21 days at five different time points. The gained data were compared to previous results using a bigger and heavier dorsal skinfold model in mice. A PubMed and a patent search were performed and all papers related to “dorsal skinfold chamber” from 1^st^ of January 2006 to 31^st^ of December 2015 were evaluated regarding the dorsal skinfold chamber models and their technical improvements. The main models are described and compared to our titanium Leipzig Dorsal Skinfold Chamber model.

**Results:** The Leipzig Dorsal Skinfold Chamber fulfils all requirements of continuous *in vivo* models known from previous chamber models while reducing irritation to the mice. Five different chamber models have been identified showing substantial regional diversity. The newly elaborated titanium dorsal skinfold chamber may replace the pre-existing titanium chamber model used in Germany so far, as it is smaller and lighter than the former ones. However, the new chamber does not reach the advantages of already existing chamber models used in Asia and the US, which are smaller and lighter.

**Conclusion:** Elaborating a smaller and lighter dorsal skinfold chamber allows research studies on smaller animals and reduces the animals’ discomfort while carrying the chamber. Greater research exchange should be done to spread the use of smaller and lighter chamber models.

## Zusammenfassung

**Einleitung:** Die Verwendung der Rückenhautkammer hat innerhalb der letzten 8 Jahrzenten wesentlich zum Verständnis der Mikrozirkulation beigetragen. Diese Technik erlaubt *in vivo* Studien der Vaskularisation über einen kontinuierlichen Zeitraum. Die Rückenhautkammer bietet eine attraktive Möglichkeit, um die Vaskularisation von autolog oder allogen transplantiertem Gewebe, der Wundheilung, der Tumorgenese oder der Kompatibilität von Biomaterialien zu untersuchen. Um das Leid der Tiere, die im Rahmen dieser Rückenhautkammermodelle Verwendung finden, zu reduzieren, haben wir ein kleineres Kammermodell entwickelt (die Leipziger Rückenhautkammer). Zudem wurde eine Übersicht der herkömmlich angewandten Rückenhautkammermodelle zusammengestellt und diese mit unserem Leipziger Modell verglichen.

**Methoden:** Die Leipziger Kammer wurde an 66 weiblichen C57Bl/6 Mäusen mit einem mittleren Gewicht von 22 g angewandt. Unter Injektion von Fluoreszein Isothiozyanid Dextran wurde die Angiogenese mit einem Axio Scope evaluiert. Die mittlere Perfusionsrate wurde über 21 Tage an fünf definierten Zeitpunkten erhoben. Die Daten wurden mit denen, welche unter Anwendung der herkömmlichen Kammer generiert wurden, verglichen. Zudem führten wir eine PubMed Recherche sowie eine Patentsuche unter Verwendung der Schlagwörter „dorsal skinfold chamber“ für den Zeitraum 1. Januar 2006 bis 31. Dezember 2015 durch und untersuchten die entsprechenden Paper auf die erwähnten Rückenhautkammermodelle sowie auf deren technischer Angaben. Die meist genutzten Modelle wurden mit dem Leipziger Kammermodell verglichen.

**Ergebnisse:** Das Leipziger Kammermodell erfüllt die Erfordernisse für Kammermodelle für kontinuierliche *in vivo* Untersuchungen, dabei erreichten wir jedoch eine deutliche Minderung des Leides der Versuchstiere. Es wurden fünf verschiedene Kammermodelle identifiziert, die deutliche Unterschiede in ihrer regionalen Anwendung finden.

Die neu entwickelte Leipziger Rückenhautkammer könnte die in Deutschland überwiegend genutzte Titan-Rückenhautkammer ersetzen, da sie kleiner und leichter ist als die herkömmliche. Allerdings bestechen Kammermodelle, die im asiatischen und amerikanischen Raum genutzt werden, mit einem noch geringeren Gewicht und niedrigerer Größe.

**Fazit:** Die Entwicklung einer kleineren und leichteren Rückenhautkammer erlaubt die Anwendung dieses Kammermodells an kleineren Tieren und mindert deren Leiden. Ein größerer Austausch über die Verwendung unterschiedlicher Kammermodelle kann dazu beitragen, die Anwendung kleinerer und leichterer Kammern zu propagieren.

## Introduction

Understanding the physiology and pathophysiology of microcirculation has always been intriguing as dysfunctional microcirculation can lead to diseases and plays an important role in inflammation. To assess microcirculation thoroughly, *in vivo* models were established. The first *in vivo* studies were done in 1882 by Kühne and co-workers using the mesenterium because of its dense vessel system [1]. Though, this tissue is not easily accessed and does not allow continuous analysis. 

Sandison was first to develop a transparent chamber to study microcirculation in the rabbits’ ear in 1929 [2] and was followed by Clark and co-workers in 1930 and later by Algire and co-workers, who adapted the model to smaller animals establishing a dorsal skinfold chamber for the mouse in 1943 [3]. Along the time, observation chambers were equally established on rats and hamsters [4], [5], [6], [7], prepared as well on the animals’ ear, cheek pouch or back [8], [9]. Since then the dorsal skinfold chamber became one of the main observation window models allowing continuous *in vivo* analysis of hemodynamic parameters such as functional vessel density and erythrocyte velocity over a period of time of about 3 weeks. In 1993 Lehr published on the use of the dorsal skinfold chamber in nude mice [10]. 

Thereafter dorsal skinfold chambers became increasingly popular. Visualizing tumours in animals and analysing various aspects of cancer physiology and vascularization, cell migration and metastasis is possible in the dorsal skinfold chamber [11], [12]. In addition, wound healing [13], thrombosis [14], [15] and volume therapy [16] studies are performed in dorsal skinfold chamber models. Currently the dorsal skinfold chamber model is broadly used in the field of biomaterial research, especially in the analysis of their biocompatibility [17], [18], [19], [20], [21], [22], [23]. 

### Potential analyses using dorsal skinfold chambers

**Figure 1:**
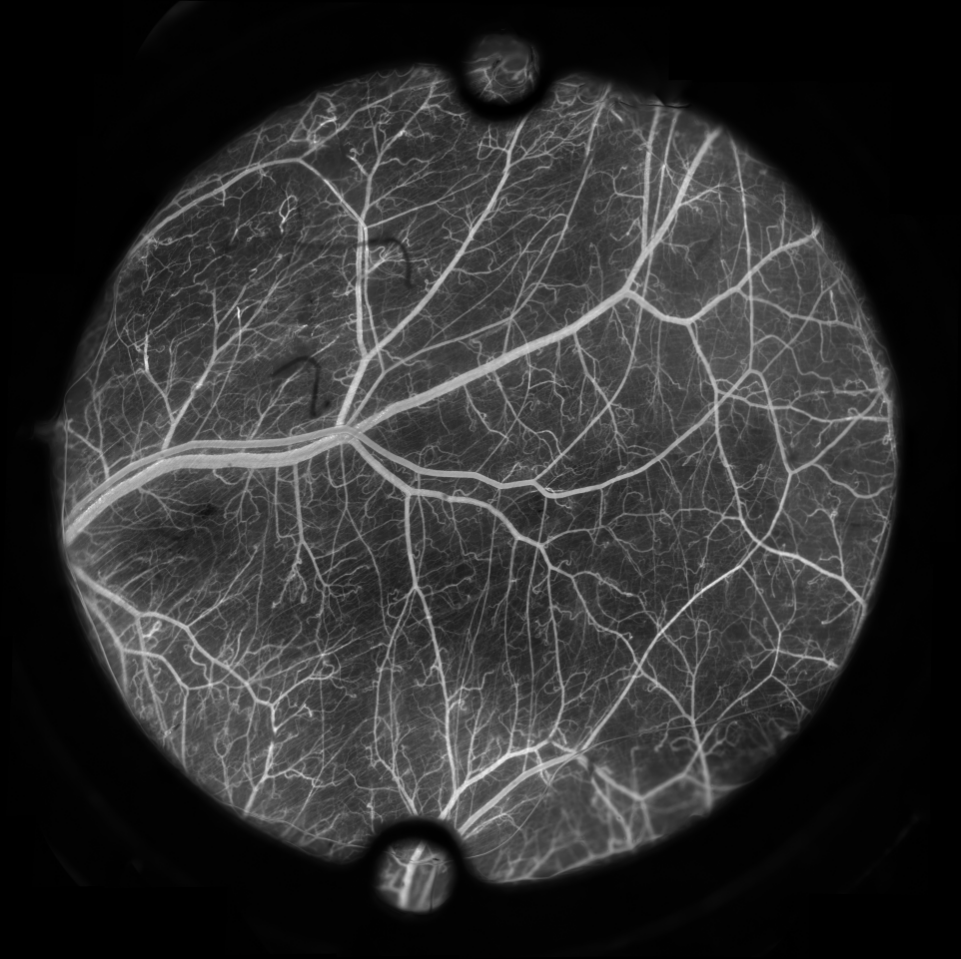
Vascularisation seen after intravenous injection of 0.5 ml FITC-labelled dextran 5% [150 kDa], 5x objective, Axio Vert Zeiss, AxioCam mR 5, Leipzig Dorsal Skinfold Chamber model

The dorsal skinfold chamber offers many possibilities for microvascular research: Repeated measurements in the same animal over a period of time of 3 weeks can be performed [8]. The observations can be done through transillumination microscopy to analyse the microvascular diameter and functional vessel density. Epi-illumination fluorescence microscopy allows for studying distinct cellular and molecular aspects by using fluorescent agents. Rhodamin 6G (100 µl of rhodamine-6-G, 0.05%, intravenously; Sigma-Aldrich, Buchs, Switzerland) illustrates the leukocyte/endothelial cell interaction by staining leukocytes. This allows for identification of rolling leucocytes, which are defined as leucocytes with lower velocity than erythrocytes [24]. Fluorescein isothiocyanate (FITC)-labelled dextran, i.e. 0.4 mg 150 kDa FITC-dextran (Sigma Aldrich, St. Louis, MO) in 100 µl PBS intravenously, depicts the vascular permeability (extravasation) and allows for visualization of blood vessels (Figure 1 (Fig. 1)). Equally, Texas Red (0.1 ml of 10 mg/ml intravenously; Invitrogen, Leek, The Netherlands) can be used to mark the blood vessels [25]. This permits blood flow analysis and vascular volume measurement, which can be done with the Velocity System Improvision (Perkin Elmer, Waltham, Massachusetts) [25].

The discovery of green fluorescent protein (GFP), and its derivatives such as blue, cyan, green, yellow, red, and far red fluorescent protein allows for creation of transgenic cell lines or animals that harbour GFP or its spectral variants under the control of the promoter of a gene of interest [26], [27]. By using these markers as a monitor system it is possible to monitor cells, tumour growth or metastasis development within the dorsal chamber. Moreover, fluorescence resonance energy transfer microscopy can be used in dorsal skinfold chambers, which permits the determination of the approach between two molecules within several nanometres. Hence, direct molecular interactions can be studied *in vivo*. Recently, Thunemann and colleagues gave an excellent description of this technique [27]. 

Furthermore, several add-ons are being developed to enlarge the portfolio of evaluation techniques with the dorsal skinfold chamber: Biel et al. introduced hyperspectral oxygen imaging within the dorsal skinfold chamber that allows for evaluation of tumour response to anti-angiogenic agents and simultaneously assesses the vascular density of the tumour and its oxygenation status [28]. Makale and his team developed a two-sided dorsal skinfold chamber with a planar oxygen sensor on one side of the skinfold to measure oxygen levels in the sandwiched subcutaneous tissue [29], [30]. Nishimura and co-workers combined the dorsal skinfold chamber technique and multiphoton laser scanning microscopy to analyse the revascularization process of pancreatic islet cells [25]. With the use of laser scanning confocal microscopy three-dimensional reconstruction of the microvascular architecture can be done [31].

In 2010 Erten and colleagues presented a new non-metallic Delrin^®^ acetal polyoxymethylene chamber model, that permits MRI chamber studies [32].

### Refinement of dorsal skinfold chambers

Given the huge increase of applications for dorsal skinfold chamber studies and the further development of inbred animal strains with low body weight [33], improving the dorsal skinfold chamber model itself in order to reduce the animals’ discomfort while carrying the chamber is a necessary goal. However, few studies are published discussing the development of smaller dorsal skinfold chambers. 

According to the three principles of refinement, reduction, and replacement of humane experimental techniques acclaimed by Russel and Burch in 1959 [34] we aimed to refine the dorsal skinfold chamber to reduce the animals’ discomfort by developing a smaller and lighter dorsal skinfold chamber model regarding manipulation, weight, proportion, biocompatibility to the regularly used model in Germany [10], [35]. Moreover, we performed a PubMed search of existing chamber models and compared them.

## Material and methods

### General technical data of dorsal skinfold chamber models

**Figure 2:**
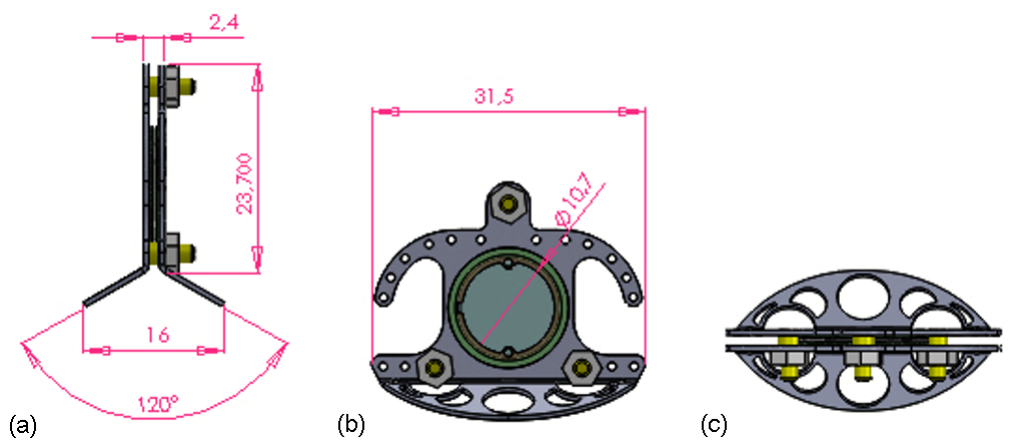
Technical representations of the Leipzig Dorsal Skinfold Chamber and its measurements. (a): lateral view, (b): frontal view, (c): view from below.

Dorsal skinfold chambers are generally made of two complementary plates sandwiching a laterally positioned fold of dorsal skin [29], [30], [35]. At the centre is an approximately 1 cm diameter circular observation area, where the skin is removed and covered by a round cover glass, that is fixed by a snap ring (Figure 2 (Fig. 2)) [35], [36].

The frames of the dorsal skinfold chamber were originally made of stainless steel or aluminium with a Teflon coating. However, the stability of aluminium, which assures a very light chamber, is not high enough to prevent bending. Furthermore, the Teflon coating is damaged with time [5]. Hence, nowadays titanium plates are widely used for dorsal skinfold chambers, satisfying the need for high rigidity as well as biocompatibility. 

### Elaboration of the Leipzig Dorsal Skinfold Chamber

**Figure 3:**
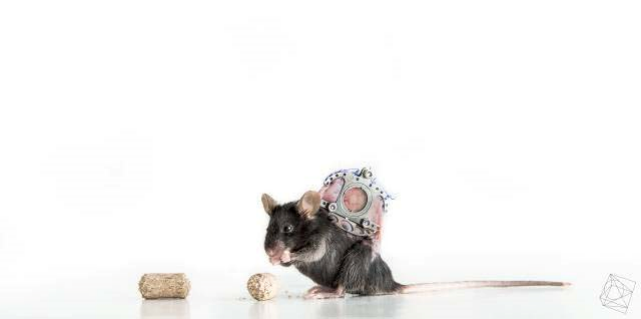
Photograph of a mouse with an inserted dorsal skinfold chamber

**Figure 4:**
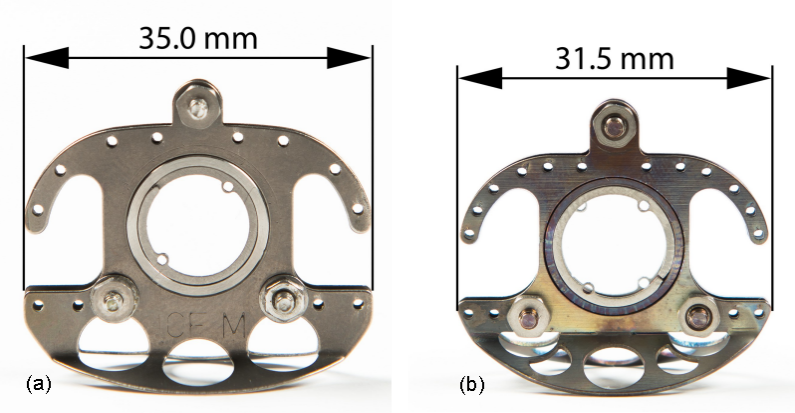
Preliminary used model (a) and recently developed Leipzig Dorsal Skinfold Chamber model for mice (b), photographs by Lieven Spur

In cooperation with the Centre of Biotechnology and Biomedicine Leipzig, a new titanium dorsal skinfold chamber (Figure 2 (Fig. 2), Figure 3 (Fig. 3), and Figure 4b (Fig. 4)) has been developed and applied to a murine C57BL/6 dorsal skinfold chamber study of angiogenesis of autologous fat transplants (licence for animal testing approved by the local governmental animal care committee of Saxony (TVV 28/13) [37]. The study was conducted in accordance with the German legislation on protection of animals and the NIH (National Institute of Health) *Guidelines for the Care and Use of Laboratory Animals*.

In comparison to the commonly used titanium dorsal skinfold chamber model for mice in Germany [10], our model is 1.5 g lighter. As the animals’ (C57BL/6, female) mean weight was 22 g, a difference of 1.5 g corresponds to 6.8% of its bodyweight. Reducing the chamber’s weight is therefore a tremendous refinement of the chamber model. Moreover, though the observation window diameter is equal to the predecessor model, the area of one titanium plate is about 28.9% smaller. As the mice’s skin is stretched to mount the chamber, a lower frame size reduces stretching of the skin and the risk of hurting the dorsal muscle.

### Mounting a dorsal skinfold chamber

To achieve best operative results, mice should have a minimal weight of 20 g. They were anaesthetized through an intraperitoneal injection with ketamine (100 mg/kg bw; Ketamine^TM^ 10%, Bela-Pharm, Vechta, Germany) and xylazine (25 mg/kg bw; Rompun^®^ 2%; Bayer Health Care, Leverkusen, Germany). The preparation was done with sterile instruments. The titanium dorsal chamber can be autoclaved. 

The mice’s backs were shaven and afterwards chemically depilated (Pilca Med; Olicia, Hamburg, Germany).

To obtain medial position of the skinfold chamber, a midline was drawn with a sterile skin pen.

The back plate of the dorsal skinfold chamber was fixed with six 4/0 polyfil resorbing sutures. Two incisions were made through the skin according to the two lower screws, so that the back plate had the right position and the dorsal skin was accurately stretched in order to correctly prepare the observation window. The animal’s side was transilluminated to define the excision site of the observation window. Preparation was done with a stereomicroscope.

The diameter of the preparation was slightly larger than the observation window diameter to prevent tissue compression and thereafter compromised blood supply [8]. Following this advice, the development of granulation tissue was diminished and the chamber lasted longer. Thereby the subcutaneous tissue, the panniculus carnosus and the retractor muscle were excised.

The preparation window was kept moist with sterile normal saline. Afterwards, the anterior chamber was fixed with titanium nuts. The space between the two retractors was about 2.4 mm. A coverslip is finally placed on the observation window and was fixed with a snap ring (Figure 3 (Fig. 3)).

For recovery of the operation, the animal was placed on a 32°C warm plate until it awoke from anaesthesia. For the following three days after dorsal skinfold chamber implantation analgesics were added to the water. No changes of eating or sleeping habits were observed in the animals. The mice’s mobility was not constrained after mounting the chamber.

### Intravital microscopy

Intravital florescence microscopy was performed using an Axio Vert microscope from Zeiss (Zeiss, Jena, Germany) with a 100 W, 12 V halogen lamp and a Zeiss filter set (BP 450–490, FT 510, LP 520) for blue and green and ultraviolet light measurements after fluorescein isothiocyanate (FITC) injection. Microscopy was performed using 5x and 20x long distance objectives from Zeiss (Zeiss, Axiotech Vario 100 HD, Acroplan 20X0.5 W, Zeiss, Jena, Germany) with a crop factor of 0.4 for the 20x magnification (Figure 1 (Fig. 1)). 

To obtain best results during intravital microscopy, the animal is sedated and placed on a Plexiglas holder, where the animal rests in a lateral position during the examination. To analyse the vascularisation 0.5 ml of FITC-labelled dextran (150,000 MG, Sigma-Aldrich) are injected into a lateral vein of the mouse-tail after disinfection. To facilitate the identification of the vein in a black 6 mouse, the tail can be incubated for 5 minutes in 30°C warm water to dilate the veins prior to injection. Immediately after successful injection the examination can be performed. 

### MEDLINE research and patent research to dorsal skinfold chamber models

We performed a PubMed search and a patent search to identify dorsal skinfold chamber models. The PubMed research with the terms “*dorsal* *skinfold* *chamber*” resulted in 368 hits. The paper examination was restricted to the years 2006 till 2016: A total of 231 papers were found and analysed. 

Among the analysed papers we identified the animal model, material of the chamber and the originating country.

The patent search was done using the German patent website [38] with the terms “dorsal skin chamber”, “dorsal skinfold chamber”, “dorsal skinfold window”, “*dorsal* *window*”, and “Rückenhautkammer”, which is the German word for “dorsal skinfold chamber”. 

## Results

### The Leipzig Dorsal Skinfold Chamber

The average operation time with autologous fat transplantation takes 28 min, the dorsal skinfold preparation without fat transplant excision lasts for 25 min. The chamber in the mouse can be used immediately and does not require a recovery day. About 8% of the operated animals show inflammation signs within the observation window, as oedema formation or vasodilatation. In some rare cases, small air pockets occur below the cover glass. In these cases, the cover glass was removed and physiological saline solution was added below the cover glass.

Microscopic analysis of the blood vessels showed a physiologically stable vascular bed within the observation window. Moderate hyperaemia was seen within the first 24 hours after the implantation but subsides within a day or two.

The back chamber model usually cannot be used longer than 3 weeks because the elasticity of the skin reduces over time and the skin shrinks, leading to a deterioration of the observation window [8].

### MEDLINE search: dorsal skinfold chamber models used in mice

Among all examined papers, 43 papers were excluded, as they did not involve mouse species. The remaining 189 papers were mouse dorsal chamber studies; 54 papers did not specify the chosen dorsal skinfold chamber model, 120 papers mentioned that the chamber model was made of titanium, 5 were made of aluminium. Only 10 papers reported using a non-metallic chamber in mice. These chambers were made of polyacetal resin, polyvinyl chloride plates, or Delrin^®^. Interestingly, studies from Germany, Austria and Switzerland, which made up 63% of all analysed studies, were all performed with titanium frames, mostly referring to the model presented by Lehr et al. or Laschke et al. [10], [35] as presented in Figure 4a (Fig. 4). American research groups also use a titanium frame (mostly from APJ Trading) [39], that is smaller than the German one. Asian researchers published on the non-metallic dorsal skinfold chamber [25], [40], [41], [42], [43]. A specific PubMed search came up with one Swedish paper dating back to 1997 presenting a Plexiglas variation of the dorsal chamber [44]. Technical data, if available, are given in Table 1 (Tab. 1).

### Patent research

Only 4 different patents were found (US020140121493A1, US020110173709A1 = WO002008108993A1, and DE 19814674 A1), but, though related to dorsal skinfold chambers, these patents do not describe a new chamber model. The German patent describes a technique to perform MRI scans using a skinfold chamber that has been used in several studies performed in Munich [45], [46]. 

## Discussion

In comparison with the other presented models, the Leipzig Dorsal Skinfold Chamber fulfils the aim of refining and reducing the animals’ discomfort carrying the chamber when compared to the model that is widely used in Germany. However, the American titanium chamber outperforms the Leipzig Dorsal Skinfold Chamber in terms of frame (area 26% smaller than the commonly used German chamber and about 10% smaller than the Leipzig model) while having a similar sized observation window.

When compared to the non-metallic models from Asia, the titanium frames are still inferior because of their higher weight and their disadvantage of not being compatible for MRI studies.

Surprisingly, no larger consent exists in the use of different dorsal skinfold chamber models. The mostly used chamber model comes along with the heaviest weight and largest frame. 

In summary, the dorsal skinfold chamber is an ideal model to study angiogenesis while satisfying the need for intravital continuous measurements, and allowing analysis of diverse tissues and textures. The long history of application of this model underlines its high value in angiogenesis and *in vitro* assessment. Refinement of this exclusive model is repeatedly done melting novelty in biotechnology and medicine. The presented Leipzig Dorsal Skinfold Chamber allows similar studies as with its predecessor model but is smaller and lighter. Nonetheless, the currently used models in Germany do not completely satisfy the requirements of refinement for animal use as described by Russel and Burch [34]. Greater scientific exchange is necessary to spread the use of the already existing smaller and lighter dorsal skinfold chamber models.

## Notes

### Competing interests

We confirm that there are no known conflicts of interest associated with this publication and there has been no significant sponsorship or funding arrangements for this work that could have influenced its outcome.

### Acknowledgement

We thank the Ingenieurbüro Hantel in Braunschweig for the cooperation for chamber development and Lieven Spur for the photographs. We also thank Mrs. Hirrlinger, Mrs. Fink-Sterba, Mrs. Weißheit, Mrs. Köbrig, Mrs. Landau and Mrs. Lemm, staff of the Medical Experimental Centre of the University Clinic of Leipzig, for taking care of the animals. 

## Figures and Tables

**Table 1:**
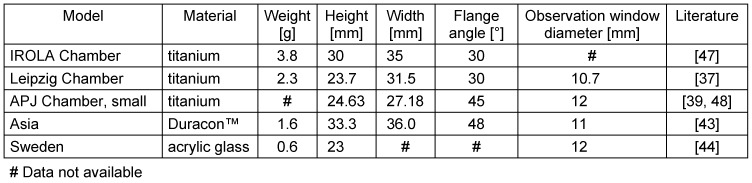
Data for different dorsal skinfold chamber models used in mice
